# Recurrent Neurobrucellosis in a Feral Swine Hunter

**DOI:** 10.7759/cureus.39383

**Published:** 2023-05-23

**Authors:** Brian Shaw, Melinda Madden, Edgar Sanchez, Steve Carlan

**Affiliations:** 1 Internal Medicine, Orlando Regional Medical Center, Orlando, USA; 2 Infectious Diseases, Orlando Regional Medical Center, Orlando, USA; 3 Academic Affairs, Orlando Regional Medical Center, Orlando, USA

**Keywords:** contaminated food, feral swine, extended treatment, zoonosis, neurobrucellosis

## Abstract

Brucellosis is a zoonotic infectious disease caused by the bacterial genus *Brucella* and is most commonly transmitted to humans globally via the consumption of contaminated unpasteurized products. In a significant minority of cases, *Brucella* has been found to be transmitted by contact with infected swine bodily fluids such as blood. Only a small proportion of all cases of brucellosis affects the central nervous system, and of the four species of *Brucella* that are known to infect humans, *Brucella suis* is unusual. Neurologic involvement occurs in a limited proportion of cases and can vary in presentation, ranging from encephalitis to radiculitis or from brain abscess to neuritis.

In this case report, we present a 20-year-old male with an eight-day history of headache and neck pain and a high fever that started two days after the onset of the headaches. Three weeks prior, he had hunted, killed, butchered, cooked, and eaten a wild boar in the field. A workup was performed, and blood cultures eventually grew *Brucella suis*. Although an intensive broad-spectrum antibiotic protocol was implemented, his post-therapy course was complicated. He eventually discontinued his antibiotics after one year.

## Introduction

Brucellosis is a zoonotic infectious disease caused by the bacterial genus *Brucella* and is commonly transmitted to humans by the consumption of contaminated unpasteurized products [1]. Less commonly, brucellosis in humans is a result of the inhalation of aerosolized particles or through skin or mucous membrane contact with an infected substance [2]. Of the four known species that cause this disease, the two most common are *Brucella melitensis* and *Brucella suis* [1,3].

Brucellosis is a significant public health concern in many parts of the world, particularly in countries with high rates of animal husbandry. Neurobrucellosis, characterized by symptoms ranging from encephalitis to focal cranial nerve deficits, is seen in only 5%-10% of cases [4]. Although there are a few reported cases of neurobrucellosis caused by *Brucella suis* in the literature, most cases are attributed to *Brucella melitensis* [5].

Here, we report the case of a 20-year-old male swine hunter who presented with symptoms of headache, neck pain, and high fever. He was ultimately diagnosed with neurobrucellosis caused by *Brucella suis*.

## Case presentation

A 20-year-old male with no significant past medical history presented in May 2020 with an eight-day history of headache and neck pain and a high fever that started two days after the onset of the headaches. The patient is a wild boar hunter who normally kills the swine himself, butchers it while in the field, grills or roasts the meat, and eats it. In interviews, he admitted that he used no protective measures (e.g., gloves) during the butchering process. The patient last hunted three weeks prior to admission.

On arrival, the patient was febrile at 103°F, and serum laboratory investigations were remarkable only for significantly increased C-reactive protein level and mildly elevated erythrocyte sedimentation rate, as seen in Table 1. A lumbar puncture revealed clear cerebrospinal fluid (CSF), and studies (Table 1) were remarkable only for elevated protein levels. The meningitis polymerase chain reaction was negative. No neck rigidity was noted on examination. Titers were ordered for the patient, and he was found to be positive for *Brucella* immunoglobulin M (IgM) and immunoglobulin G (IgG).

**Table 1:** Laboratory values including the subsequent 11 months.

Laboratory Values		
Name	Value	Reference range
Serum white blood cell count	7.7 × 10^9^/L	4.5 × 10^9^/L to 11.0 × 10^9^/L
Serum C-reactive protein (CRP)	38.7 mg/L	<9.9 mg/L
Serum erythrocyte sedimentation rate (ESR)	16 mm/hour	0-15 mm/hour
Cerebrospinal fluid protein	80 mg/dL	15-45 mg/dL
Cerebrospinal fluid glucose	64 mg/dL	40-70 mg/dL
Serum immunoglobulin G (IgG) four months post initial infection	1:320	
Serum immunoglobulin G (IgG) seven months post initial infection	1:160	
Serum immunoglobulin G (IgG) 11 months post initial infection	1:160	

The patient was positive for *Brucella* IgM and IgG, and blood cultures eventually reported *Brucella suis*. While an inpatient, he was treated with doxycycline and gentamycin. The patient was discharged on a treatment regimen of ciprofloxacin twice daily and gentamicin daily for 10 days, ceftriaxone twice daily for four weeks, and oral doxycycline twice daily for 45 days, which was completed. The patient was doing well until four months after the original admission when he returned to the hospital again complaining of headaches, neck pain, and high fevers. Blood cultures were drawn again and revealed *Brucella suis* once more. Magnetic resonance imaging and echocardiogram were negative for lesions or vegetations. CSF cultures were negative. The patient was discharged on ceftriaxone, gentamicin, and doxycycline. Rifampin was not able to be started at that time because of transaminitis. The patient reported compliance with this regimen. After completing the intravenous (IV) antibiotics, the patient was started on oral doxycycline and oral sulfamethoxazole and trimethoprim. He was given monthly follow-up appointments for the outpatient clinic.

The patient had a nuclear medicine scan six months after his initial diagnosis that showed prominent bilateral inguinal lymphadenopathy and lymph node white blood cell (WBC) activity. A follow-up nuclear medicine scan eight months after the initial diagnosis showed decreased WBC accumulation in those same areas, indicating the improvement of his infection. *Brucella* titers were checked intermittently over 11 months and remained positive (Table 1). The patient stopped taking the medications after one year of treatment and continues to feel well, without headaches, vision changes, neck pain, fevers, or chills. The patient still goes swine hunting with his friends but no longer handles the meat. The patient now avoids eating swine meat in general.

## Discussion

The patient butchered the swine in the field and cooked the meat. During the process, his skin and mucous membranes were exposed to the contaminated swine blood. Nonetheless, it is not possible to determine with certainty if he became infected by the inhalation of aerosolized particles, consumption of infected meat, or skin and mucous membrane exposure to blood [1,2].

Clinically, brucellosis is characterized by spiking fevers, malaise, headache, and abdominal pain of variable incubation time [1,4]. Virtually, all organ systems can eventually become involved, including the cardiac, pulmonary, ocular, and dermal systems. Joints become infected in up to 80% of cases, the genitourinary system in up to 10%, and the nervous system in up to 5%-10% [6]. The clinical presentation of neurobrucellosis varies widely. In our patient, it presented as subacute meningitis; however, presentations can range from encephalitis to radiculitis or from brain abscess to neuritis [6]. The diagnosis of infected individuals can be problematic. Molecular testing is anticipated but currently not available for routine use and cannot be used to identify the species [7]. A biopsy of involved tissue may show a nonspecific granulomatous pathology [1]. Diagnosis can be established with automated blood culture systems in only 70% of cases at best [8]. The interpretations of serologic testing are highly variable and depend on multiple factors, including the length of time the infection has been present, whether the tested person is from a population where *Brucella* is endemic and already has antibodies, and the fact that there are other microorganisms that can cross-react.

This patient was a habitual feral swine hunter, which increases the chances that he would have already demonstrated *Brucella* antibodies. In addition, there is a chance that a false negative can occur [1,3]. Nonetheless, while there may be differing opinions, serologic testing for the presence of antibodies has become the gold standard of care when used in the context of clinical presentation [9]. Neurobrucellosis treatment usually consists of ceftriaxone for 4-6 weeks and rifampin and doxycycline for at least 12 weeks. As the patient in this case was unable to be started on rifampin due to transaminitis, ciprofloxacin and streptomycin were added. Additionally, relapse is common within the first six months of treatment, with a relapse rate of 5%-15%, which is treated first by repeating the initial regimen, as relapse associated with antibiotic resistance is rare [10]. Subsequent relapses warrant the consideration of an alternative antibiotic choice [10]. Despite their rarity, these cases are becoming increasingly common. Therefore, it is important that both a gold standard of diagnostic workup and guidelines are established, as these would ensure that optimal medical therapy can be started as soon as possible, which may mitigate complications associated with delayed diagnosis. A high level of awareness is necessary when encountering this patient population given their unique exposure and the endemic nature of *Brucella* in feral swine.

## Conclusions

 There are four main conclusions to this case. First, although neurobrucellosis is rare, clinicians should retain a high index of suspicion and obtain complete social histories from patients, particularly in regions where *Brucella* is endemic to feral swine. In addition, the minority of neurobrucellosis cases are caused by *Brucella suis*. Second, the diagnosis of brucellosis can be difficult because molecular testing is currently not available for routine use, and *Brucella* grows very slowly on blood cultures, if at all. Serologic testing for the presence of antibodies has become the standard of care when used in the context of clinical presentation. Third, multiagent antibiotic protocols are the therapy of choice. Since relapse is common, compliance with the protocol would seem to be a critical element of success. Fourth, brucellosis remains a risk to humans in contact with animals, and protocols to prevent disease transmission should be taught and practiced.
